# Dual Ionic and Organic Nature of Ionic Liquids

**DOI:** 10.1038/srep19644

**Published:** 2016-01-19

**Authors:** Rui Shi, Yanting Wang

**Affiliations:** 1 State Key Laboratory of Theoretical Physics, Institute of Theoretical Physics, Chinese Academy of Sciences, 55 East Zhongguancun Road, P. O. Box 2735, Beijing, 100190 China

## Abstract

Inherited the advantages of inorganic salts and organic solvents, ionic liquids (ILs) exhibit many superior properties allowing them promising green solvents for the future. Although it has been widely acknowledged that the unique features of ILs originate from their dual ionic and organic nature, its microscopic physical origin still remains blurry. In this work, by comparing the ion/molecule cage structures obtained from molecular dynamics simulations for seven prototypic liquids—a molten inorganic salt, four ILs, a strongly polar organic solvent, and a weakly polar organic solvent, we have revealed that the depth of the cage energy landscape characterizes the ionic nature of ILs, whereas the slope and curvature of its mimimum determine the organic nature of ILs. This finding advances our understanding of ILs and thus will help their efficient utilization as well as the systematic design of novel functionalized ILs.

The establishment and development of modern industries cannot be apart from creation and application of novel liquids. Featured by many advantageous properties including good tunability and solvation ability, organic solvents have been widely used in daily and industrial activities. However, due to their organic nature, organic solvents are usually volatile, which greatly raises the risk of flammation and pollution^1^. To overcome these problems, inorganic molten salts working at high temperatures were once regarded as a “green” replacement to organic solvents in many applications^2^. Despite their many advantages, such as non-flammability, non-volatility, good stability, and conductivity, inorganic molten salts suffer from the high melting temperature owing to their strong ionic nature^3^. The breakthrough did not appear until a series of air- and water-stable organic salts whose melting point is around room temperature, better known as ionic liquids (ILs), were successfully synthesized in 1992^4^. Inherited both the organic nature of organic solvents and the ionic nature of inorganic salts, ILs have even more advantageous properties, such as good solvation ability and tunability, low melting temperature, good conductivity, wide electrochemical window, thermal and electrochemical stabilities, non-volatility, and non-flammability^5^,^6^, which allow them to be promising “green” solvents utilized in many areas, such as chemical synthesis^5^,^7^, catalysis^5^,^8^, energy storage^5^,^9^, lubricants^5^,^10^, materials^5^,^11^, and biology^5^,^12^. Even though not all the ILs discovered so far are toxicity-free and biodegradable, their non-volatile and non-flammable features greatly reduce the risk of possible pollution and danger. Since different combinations of cation and anion produce at least millions of available ILs (much more than organic solvents)^5^ with a large variety of physical and chemical properties, one can in principle always find a suitable candidate to meet the requirement of a designated application. On the other hand, also because of the vast number of candidates, determining the properties of all ILs one by one by experiment is unfeasible. Therefore, selecting suitable candidates to meet specific requirements by computer-aided systematic design is essential for the efficient and smart utilization of ILs. A profound understanding of the dual ionic and organic nature of ILs is one of the key problems for fulfilling this goal.

It is well acknowledged that strong long-range electrostatic interactions characterize the ionic nature of inorganic molten salts^13^, and comparatively strong short-range van der Waals (VDW) interactions feature the organic nature of organic solvents^14^. In contrast, although the mixed ionic and organic nature of ILs has been widely discussed in literature^15^,^16^,^17^, their microscopic physical origin characterized by interionic interactions still remains unclear. In recent years, great efforts have been made to measure the interionic interactions in ILs by various spectroscopy techniques. By using the far-infrared spectroscopy, Ludwig *et al*.^18^ studied the low-frequency vibrational spectra of several ILs and related them to the interionic force constant by taking the harmonic assumption. Shirota *et al*.^19^ measured the low-frequency spectra of IL 1-methoxy-ethylpyridinum dicyanoamide by femtosecond optical heterodyne-detected Raman-induced Kerr effect spectroscopy and compared with its analogous isoelectronic neutral solution composed of 1-methoxyethylbenzene and dicyanomethane. On the basis of the observation that the IL had a higher-frequency interionic vibrational mode, they concluded that ILs have a larger intermolecular force constant than neutral solutions. By performing vibrational Stark effect spectroscopy experiments, Zhang *et al*.^20^ measured the intrinsic electric fields in several ILs and found that they are only slightly stronger than but still comparable to those in common organic solvents, which challenges the stereotype that ILs have much stronger electrostatic interactions than organic solvents.

Besides spectroscopy experiments, interionic interactions in ILs have also been studied by computation and simulation. The electronic structure and density functional theory (DFT) methods have been used to calculate the ion-pair binding energy in ILs. For instance, Tsuzuki *et al*.^21^ calculated the ion-pair binding energies of several ILs with the MP2 method and found that the electrostatic interaction is the major source of attraction between an ion pair, while the induction (part of VDW interactions) contribution is small but not negligible (around 9–13%). Kirchner and coworkers^22^ studied the ion-pair binding energies of NaCl and 1,3-dimethylimidazolium chloride with the HF and MP2 methods. They reported that the VDW (both dispersion and induction) interactions play an important role in determining the energy landscape of IL ion pairs, whereas in NaCl, the VDW contribution is almost negligible. Those calculations have successfully differenciated the contributions from the electrostatic and VDW interactions, which is of great importance for analyzing the ionic and organic nature of ILs. However, limited by computer power, those studies can only be conducted for an ion pair or small clusters in the gas phase, leaving behind a profound understanding of the microscopic origin of the dual ionic and organic nature of ILs in the condensed phase.

It is well known that many ILs with intermediate side chains form spatially heterogeneous structures in which alkyl chains aggregate to form non-polar domains and the charged groups form a continuous ionic network^23^,^24^,^25^,^26^,^27^,^28^. If we further increase the side chain length, those ILs may form various ionic liquid crystals^29^,^30^,^31^,^32^. In these structures, the IL properties also become spatially heterogeneous: it is more “ionic” in the ionic network, but more “molecular” in the non-polar domains. Therefore, we believe that, similar to short-chain ILs, ILs with intermediate chain lengths also exhibit the intrinsic dual nature determined by molecular structures and interactions, which is the central focus of this paper.

In this work, by performing all-atom MD simulations, we calculated in detail the interionic interactions in four typical ILs (1-butyl-3-methylimidazolium nitrate ([BMIM][NO_3_]), 1-butyl-3-methylimidazolium tetrafluoroborate ([BMIM][BF_4_]), 1-butyl-3-methylimidazolium hexafluorophosphate ([BMIM][PF_6_]), and 1-butyl-3-methylimidazolium bis(trifluoromethylsulfonyl)imide ([BMIM][NTf_2_])), and compared with a typical molten inorganic salt (molten sodium chloride, NaCl), a strongly polar liquid (Dimethyl sulfoxide, DMSO), and a weakly polar liquid (toluene) (Fig. 1). The obtained forces, vibrational force constants, intrinsic electric fields, cohesive energies, and cage energies for those liquids allowed us to depict the dual ionic and organic nature of ILs from the viewpoint of the cage energy landscape (CEL). The mechanism we have revealed is that the depth of the CEL determined by the long-range electrostatic interactions characterizes the ionic nature of ILs, whereas the slope and curvature of the CEL determined by the short-range VDW interactions describe the organic nature of ILs.

## Results

### Physical properties

As a novel “green” solvent with many unique properties, ILs have attracted a lot of attentions from both scientific and industrial communities for about twenty years. Therefore, many experimental data are now available for ILs. In Table 1, we list the experimental values and our simulation results for some physical properties of the seven prototypic liquids. It can be seen that our calculated densities and dipole moments agree well with the experimental values. In the following sections, we will also show that our simulation results qualitatively reproduce the experimental measurements of electric field and heat of vaporization. These results manifest the reliability of the AMBER force field and our simulation procedure described in the Methods section.

Despite their ionic nature, ILs still in some ways behave closer to organic solvents than to molten inorganic salts. As shown in Table 1, molten NaCl has a much higher melting temperature than the other six liquids due to its high cohesive energy induced by its strong electrostatic interactions. All ILs remain in the liquid state at room temperature, similar to organic solvents. ILs have much higher densities than toluene, but are still in between molten NaCl and DMSO. Molten NaCl has the highest density in the seven liquids due to its heavy inorganic ions and strong electrostatic attractions. Unlike molten NaCl, the ILs show much smaller surface tensions very close to DMSO, and almost falls into the range between DMSO and toluene, consistent with the surface tension calculations by Weiss *et al*.^48^, who showed that the reduced surface tension of ILs follows the universal scaling curve for polar organic solvents rather than inorganic molten salts.

Despite the similarities, ILs have unique properties that distinguish them from organic solvents. For instance, it can be seen from Table 1 that all the ILs have higher viscosities and smaller diffusion coefficients than the organic solvents by several orders of magnitudes (NaCl has a very fast dynamics due to the high simulation temperature of 1148.15 K to allow it in the liquid state). Except [BMIM][BF_4_] that has been simulated by a reduced-charge model, the dynamics of other ILs are significantly underestimated, because the molecular models employ full ionic charges, which omits the charge-transfer and polarizability effects. Other advantageous features of ILs not listed in Table 1 include negligible vapor pressure, tunable solvation, good conductivity, and distinctive catalytic ability^5^,^6^. In the following sections, the molecular origins leading to the differences of those physical properties will be interpreted by a detailed analysis of intermolecular interactions. From now on “molecule” will be frequently used to refer to both “ion” and “molecule” without differentiation.

### Intramolecular charge distributions

**Figure 2:**
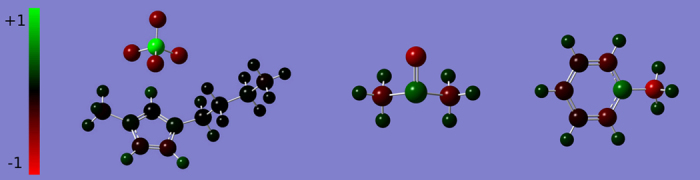
Charge distributions. From left to right: [BMIM][BF_4_] ion pair, DMSO and toluene molecules. The color bar is displayed at the left. More green colored atoms have more positive partial charges, more red ones have more negative partial charges, and black ones are almost neutral. Charge delocalization was observed in [BMIM][BF_4_], DMSO, and toluene, which enhances the electrostatic interactions in polar organic solvents but weakens the electrostatic interactions in ILs.

Intramolecular charge distribution and corresponding electrostatic interaction undoubtedly play an important role in determining the physicochemical properties of ionic systems. Inorganic salts usually consist of two or more kinds of inorganic ions with integer charges, inside which a single ion does not have an inner charge distribution (given that we ignore the induced polarization). In contrast, the effective partial charges of IL molecules are delocalized among different atoms, similar to polar organic solvents. The charge distributions of [BMIM][BF_4_], DMSO, and toluene, displayed in Fig. 2, were taken from refs 49, 50, 51, respectively. It can be seen that the charges are widely distributed among [BMIM] and [BF_4_] atoms, and only a few atoms, such as the hydrogen atoms on the imidazolium ring (+0.2 *e*) and the fluorine atoms (−0.4 *e*), have large partial charges. To effectively incorporate the charge transfer effect^52^, all the partial charges for [BMIM][BF_4_] were rescaled by 0.807^49^. Organic solvents have similar charge delocalization, despite the fact that the molecules in whole are charge neutral. The charge distribution in DMSO results in a very large dipole moment of 3.96 D, whereas in toluene the dipole moment is only 0.35 D. Generally speaking, the delocalization of charges effectively enhances the electrostatic interactions in polar organic solvents, but weakens the electrostatic interactions in ILs along with the charge transfer effect.

### Liquid structures

**Figure 3:**
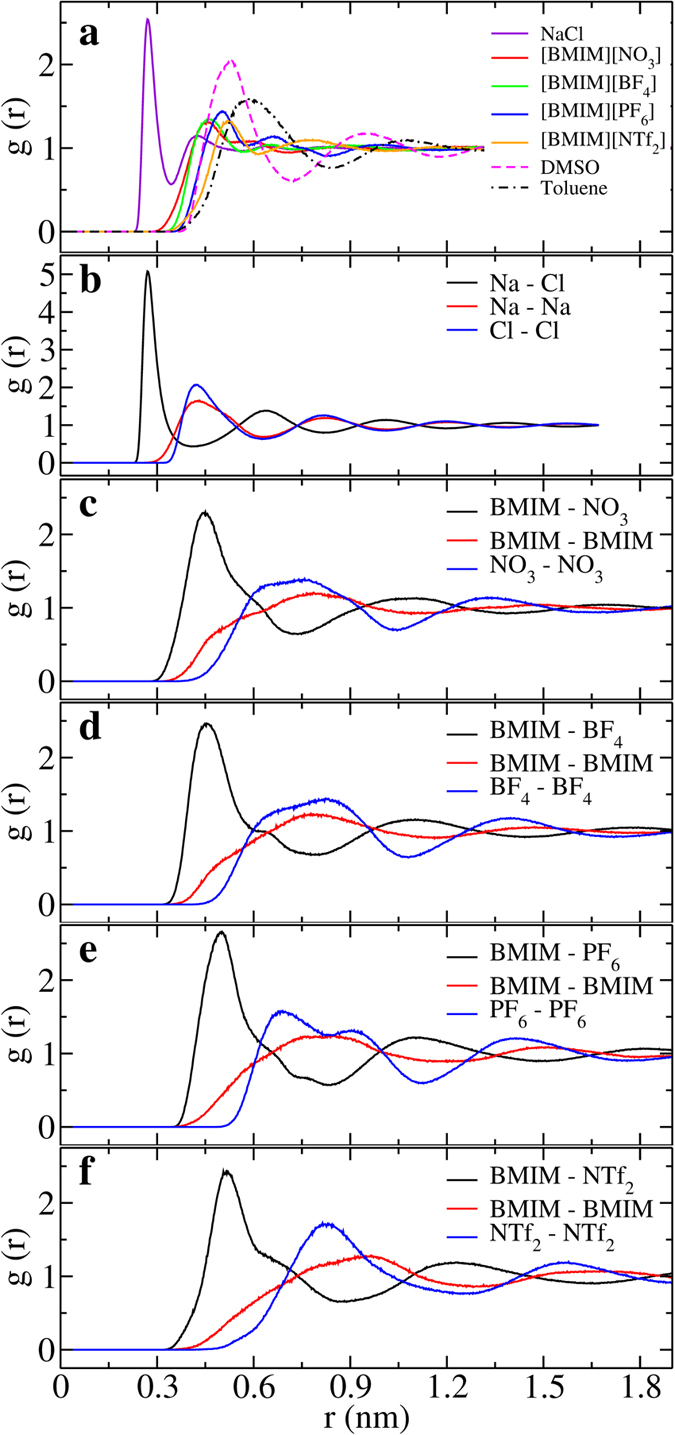
Center-of-mass radial distribution functions (COM-RDFs). (**a**) COM-RDFs of molten NaCl, [BMIM][NO_3_], [BMIM][BF_4_], [BMIM][PF_6_], [BMIM][NTf_2_], DMSO, and toluene. For ionic systems, cations and anions are treated identically in this plot. Because of the large volume of organic ions, the ion-ion distances in the first coordination shell of ILs (4.5 Å for [BMIM][NO_3_], 4.6 Å for [BMIM][BF_4_], 5.0 Å for [BMIM][PF_6_], 5.2 Å for [BMIM][NTf_2_]) is much farther than that in molten NaCl (2.7 Å), but slightly closer than those in DMSO (5.3 Å) and toluene (5.9 Å). (**b**) COM-RDFs for the cation-anion, cation-cation, and anion-anion pairs in molten NaCl. The valley positions of the cation-anion RDFs perfectly match up with the peak positions of cation-cation and anion-anion RDFs, suggesting an ordered ion cage structure. (**c–f**) COM-RDFs for the cation-anion, cation-cation, and anion-anion pairs in (**c**) [BMIM][NO_3_], (**d**) [BMIM][BF_4_], (**e**) [BMIM][PF_6_] and (**f**) [BMIM][NTf_2_]. Due to the large volume and asymmetric geometry of organic ions, the valley positions only approximately match up with the peak positions, indicating a less ordered ion cage structure in the IL.

The center-of-mass radial distribution functions (RDFs) of the seven liquids are shown in Fig. 3. In order to compare with organic solvents, in Fig. 3a, the cations and anions in NaCl and ILs are treated as identical particles in the RDF calculations. The first peak positions, characterizing the ion-ion distance in the first coordination shell, have the order NaCl (2.7Å) < [BMIM][NO_3_] (4.5 Å) < [BMIM][BF_4_] (4.6 Å) < [BMIM][PF_6_] (5.0 Å) < [BMIM][NTf_2_] (5.2 Å) < DMSO (5.3 Å) < Toluene (5.9 Å), which can be understood as follows. Due to the small volume and strong electrostatic interaction, ions in molten NaCl are closely packed in the first coordination shell. Because of the large volume of organic ions, the distances between ions in the first coordination shell of ILs are much larger than NaCl, but still slightly closer than organic solvents. The distance between DMSO molecules is closer than toluene because of its strong intermolecular interactions resulted from the large dipole moment.

**Figure 4:**
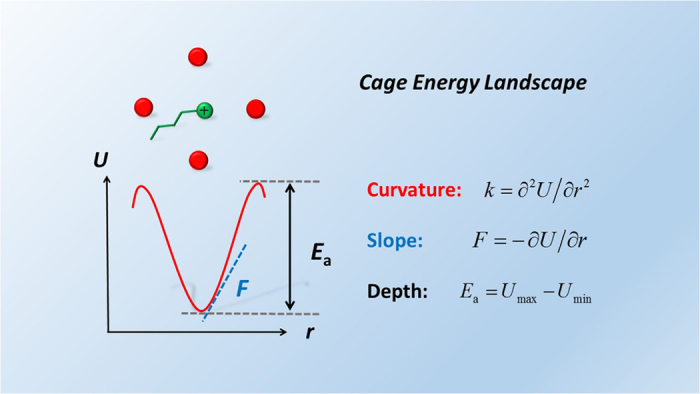
cage energy landscape. The cage energy landscape is characterized by three parameters: curvature, slope, and depth, corresponding to the force constant, force, and activation energy experienced by molecules, respectively.

Figure 3b–f show the RDFs of molten NaCl and ILs, respectively, with cations and anions treated differently. Owing to the large volume and asymmetric geometry of organic ions, all the ILs show larger ion-ion distances and lower degree of charge ordering than molten NaCl, which along with the charge delocalization, greatly reduces the electrostatic interactions and thus stands out the organic nature of ILs. Moreover, the cation-cation and anion-anion RDFs oscillate out of phase with the cation-anion RDFs, indicating a local charge ordering that extends to several coordination shells in the ionic systems. As a result, each ion is surrounded by several counterions in the first coordination shell, forming an *ion cage*^53^,^54^,^55^,^56^,^57^,^58^,^59^,^60^,^61^ composed of the counterions in the first coordination shell surrounding a central ion (see the schematic in Fig. 4). The *cage* concept can also be easily applied to molecular liquids, in which each molecule is encapsulated by a *molecule cage*^62^,^63^,^64^,^65^,^66^.

The *cage structure* averaging over all local cage structures statistically depicts the structure and dynamics of liquids. For instance, a smaller cage volume corresponds to a higher density, and a more stable cage leads to a slower dynamics. We then further define the associated *cage energy landscape* as the ensemble-averaged local energy landscape as a function of the dislocation of the central ion from the cage center. The structural and dynamical properties of liquids can be related to the CEL as: molecules vibrate near the minimum of the CEL and frequently escape the cage to diffuse. Due to its collective nature, direct determination of the CEL by experiment or simulation is still challenging. Instead, in this work, we use three parameters, namely the curvature and slope near the minimum and the depth, to characterize the main features of the CEL. Under the harmonic approximation, the curvature, slope, and depth of CEL are in fact the force constant 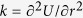, force 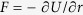, and activation energy 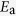 experienced by molecules, as illustrated in Fig. 4. Here the activation energy is defined as the average energy of a particle required to climb over the energy barrier and escape the cage.

### Vibrational Force constants

Many vibrational spectroscopy experiments were performed to determine the intermolecular interactions in liquids. For example, as mentioned in the Introduction, Shirota *et al*.^19^ found that an IL has the interionic vibrational frequency about 20% higher than its isoelectronic molecular solution, suggesting that ILs have a slightly larger vibrational force constant than organic solvents. However, this method is not always reliable, since it is unfair to directly compare the frequencies that may belong to different vibrational modes. To avoid this problem, we employ the first moment of vibrational density of state (VDOS) to qualitatively describe the average characteristic frequency of intermolecular vibrational modes, which is defined as^67^ 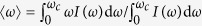, where 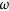 is the frequency and 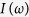 is the VDOS, the Fourier transform of the velocity time autocorrelation function. The frequency 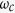 was chosen in such a way (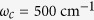 for NaCl and 175 cm^−1^ for other liquids) that all the intramolecular vibrational modes were excluded and the main intermolecular modes were included. The calculated VDOS, shown in Figure S1 in the Supplementary Information (SI), agrees well with the optical heterodyne-detected Raman-induced Kerr effect spectra^68^,^69^,^70^.

The vibrational force constants in liquids, listed in Table 2, were calculated under the harmonic approximation, 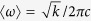, where *c* is the speed of light and 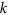 is the mass-scaled force constant. It can be seen that the characteristic frequencies (first moments) calculated for the ILs and the organic solvents agree very well with the experiments (no experimental data are available for molten NaCl and [BMIM][NO_3_]). Clearly, the characteristic frequencies and force constants of ILs are close to polar liquid DMSO, but much smaller than molten NaCl. These results suggest that the curvature of the IL CEL is similar to polar organic solvents but significantly gentler than inorganic molten salts.

### Forces

In an equilibrium state, the long-time average of the instantaneous force experienced by a molecule is zero, but its instantaneous magnitude fluctuates with time, reflecting the strength of the intermolecular force in liquids. To make the effect of interactions on dynamics comparable for different systems, the force is scaled by particle mass, which is actually the instantaneous acceleration.

**Figure 5:**
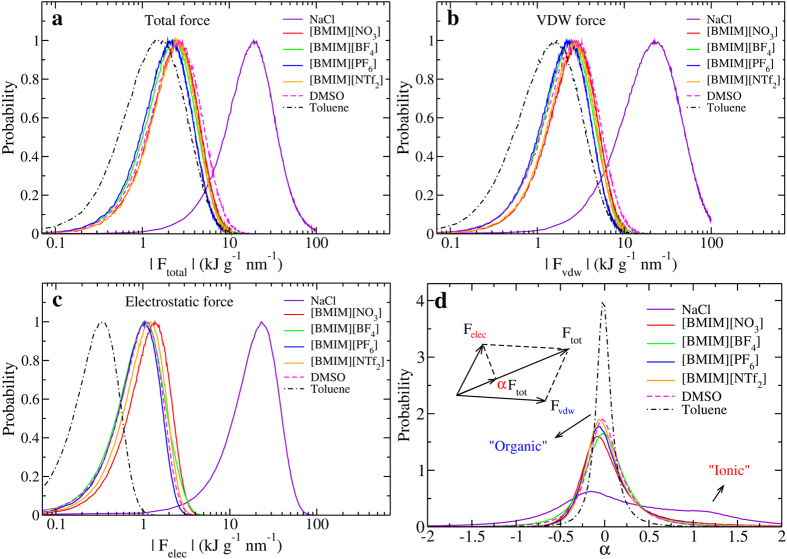
Distribution of intermolecular forces. (**a**) Total, (**b**) VDW, and (**c**) electrostatic interactions in molten NaCl, [BMIM][NO_3_], [BMIM][BF_4_], [BMIM][PF_6_], [BMIM][NTf_2_], DMSO, and toluene. The *X* axis is displayed in the log scale and the *Y* axis is in an arbitrary unit. (**d**) Contribution of electrostatic force to the total force. This contribution is characterized by a parameter 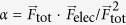. The peak positions at around *α* = 0 suggest that the VDW interaction dominates the intermolecular force in ILs and organic solvents. The width of the *α* distribution (normalized by area) describes the organic and ionic nature of forces in liquids—narrower means more organic and wider means more ionic.

Figure 5a–c compare the total, VDW, and electrostatic forces, respectively, in all liquids. It can be seen from Fig. 5a that the DMSO molecule experiences stronger intermolecular force than toluene because of its larger dipole-dipole interaction. The forces experienced by ions in ILs fall in the range between DMSO and toluene, but one order of magnitude weaker than molten NaCl. Particularly, the electrostatic and VDW forces in ILs are nearly as strong as DMSO, and falls in the range between NaCl and toluene.

The electrostatic force in molten NaCl is comparable to the VDW and total forces, whereas in other liquids, the electrostatic forces are significantly weaker. We further define a parameter 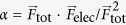 to characterize the electrostatic contribution to the total intermolecular force, whose schematic is shown in the inset of Fig. 5d. The parameter 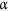 quantifies the ionic feature by the intermolecular force which is independent of particle mass, size and number of interacting sites per molecule. It can be seen from Fig. 5d that, except molten NaCl, all liquids show a single peak at around 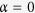, demonstrating that the intermolecular force is dominated by the VDW interaction in ILs and organic solvents. Molten NaCl shows two peaks at −0.2 and 1.2, demostrating the significant role of electrostatic force in molten NaCl. Moreover, the width of the distribution increases in the order: toluene < DMSO ~ ILs < NaCl, indicating an increasing electrostatic contribution to the intermolecular force as the liquid changes from more organic to more ionic. Interestingly, all the ILs show a distribution surprisingly closer to DMSO, , suggesting the organic features of ILs, consistent with our force constant result shown above. In contrast, both Na and Cl ions have much wider distributions with the peak position deviated from zero, reflecting the strong inorganic and ionic nature of inorganic molten salts. Along with the results of force constants, we conclude that the curvature (estimated by force constant) and slope (deterimined by force) of the IL CEL are mainly determined by the VDW interactions, similar to polar organic solvent, which underlines the organic nature of ILs.

### Intrinsic Electric fields

In our previous work^20^, the vibrational Stark effect spectroscopy experiment demonstrated that the intrinsic electric fields in ILs fall in the range between DMSO and tetrahydrofuran. Here we calculate directly from our simulations the electric fields experienced by molecules in all the liquids studied in the present paper. It can be seen from Table 2 that, in good agreement with the experimental measurement, the intrinsic electric fields in ILs is in the range between DMSO and toluene and about four times weaker than in molten NaCl. This result again supports our conclusion that the strength of the electrostatic interaction in ILs is comparable to polar organic solvents, but much weaker than in molten inorganic salts, whose reasoning is as follows. On one hand, the large volume, asymmetric geometry, and charge delocalization of organic ions effectively weaken the electrostatic force in ILs. On the other hand, the charge distribution of polar (neutral) molecules significantly enhances the electrostatic interaction in organic solvents.

### Cohesive energies

The heat of vaporization, the energy required to transform a system from the liquid to the gas phase, can be calculated by


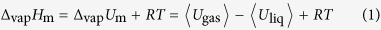


where *R* is the gas constant, *T* is the temperature, 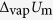 represents the cohesive energy, and *U*_gas_ and *U*_liq_ are the molar internal energies in the gas and liquid phases, respectively. Because almost all ions form pairs in the gas phase^72^, *U*_gas_ is replaced by the molar internal energy of an isolated ion pair in molten salts and ILs. Therefore, the cohesive energy in ionic fluids actually measures the energy required to transform a condensed ionic fluid (liquid phase) to isolated ion pairs (gas phase). As shown in Table 3, the calculated heat of vaporization data agree well with the experimental results. Note that 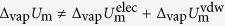 due to the slight difference coming from the intramolecular interactions. As expected, the cohesive energy and its electrostatic contribution apparently increase as the liquid changes from more organic to more ionic. The electrostatic part dominates the cohesive energy in molten NaCl and the VDW part dominates in toluene, but the two parts are comparable in the ILs and DMSO. Despite the omission of the charge-transfer and polarization effects for [BMIM][NO_3_], [BMIM][PF_6_], and [BMIM][NTf_2_], our calculated Coulomb interaction contributes 46% for [BMIM][NTf_2_], in good agreement with the value of 41% reported by Rebelo *et al*.^73^ at 298.15 K. Again, the ILs have the electrostatic contribution closer to the polar organic solvents than the molten inorganic salt. This result can be interpreted by a conceptual “ion pair” view of ILs: within the “ion pair” life time, ILs can be regarded as strongly polar “molecular” liquids composed of temporary neutral “ion pairs”. At a time scale longer than the ion-pair life time, “ion pairs” break and ions diffuse to other positions to form new “ion pairs”. This picture may also interpret the similarities in some static properties and short-time behaviors between ILs and organic solvents. However, this picture is rather sketchy since ions in ILs are usually coordinated with several counterions, rather than bonded with a specific one. Therefore, in next section, we will better interpret the interactions in ILs from the “ion cage” viewpoint, which quantitatively depicts the ionic nature of ILs.

### Cage energies

Instead of forming ion pairs in the gas phase, ions in ILs form ion cages in the liquid state (see the schematic in Fig. 4). If we define an “ion pair” in the liquid state as composed of an ion and its nearest counterion, it is not surprising that this ion pair has a larger distance than in the gas phase due to the many-body effect (see Fig. S2 in the SI). Therefore, the previous studies on the binding energy of an IL ion pair can hardly describe the real ion-ion interactions in the liquid phase. Instead of the gas-phase binding energy, we define a novel liquid-phase *cage energy* 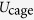 as the average potential energy between an ion and a counterion in its ion cage to characterize the local ion-ion interaction in the liquid.

The cage energies and their electrostatic and VDW contributions have been calculated from MD trajectories and are summarized in Table 4. The cage energy for [BMIM][BF_4_] is much lower than the ion-pair binding energy in the gas phase due to the loose packing in the condensed phase (see the SI for the details). The cage energy apparently decreases as the liquid changes from more organic to more ionic, mainly attributed to the enhanced electrostatic interaction. In particular, from DMSO to ILs, the electrostatic energy drops from −7.2 to around −200 kJ/mol, indicating that ILs have a much more stable cage structure than organic solvents. In contrast, all the ILs have similar attractive VDW energy as in the two organic solvents, but NaCl has a repulsive VDW energy. This is because small inorganic ions closely contact with each other due to strong electrostatic attractions, whereas organic ions in ILs are kept farther by their large volume and asymmetric geometry, as demonstrated in Fig. 3.

Based on the roles of electrostatic and VDW interactions in determining the cage energy for the seven liquids, the dual ionic and organic nature in ILs, compared with inorganic salts and polar liquids, can be interpreted as the following:

1. weakly polar liquid (e.g. toluene): the VDW interaction dominates the cage energy;
2. strongly polar liquid (e.g. DMSO): the electrostatic and VDW interactions contribute almost equally to the cage energy;
3. ionic liquid (e.g. [BMIM][NO_3_], [BMIM][BF_4_], [BMIM][PF_6_] [BMIM][NTf_2_]): the electrostatic interaction dominates the cage energy (***ionic nature***) and the VDW interaction is attractive and has a similar strength as in organic solvents (***organic nature***);
4. inorganic molten salt (e.g. NaCl): the electrostatic interaction dominates the cage energy (ionic nature) and the VDW interaction is repulsive (inorganic nature).

### Mechanism

**Figure 6:**
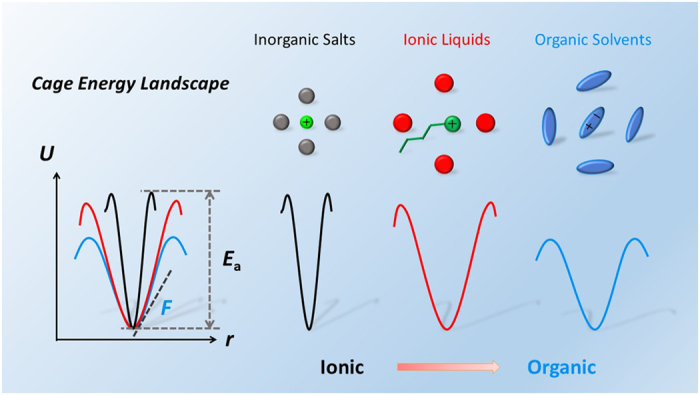
Cage energy landscape. Schematic illustration of cage structures and cage energy landscape in inorganic salts, ionic liquids, and organic solvents. The cage energy landscape of inorganic salts is deep and steep, whereas that of ionic liquids is still deep but much more gently. Organic solvents and ionic liquids have a similar slope and curvature near the minimum of the cage energy landscape, but the depths for the organic solvents are much lower.

Organic ions have comparable VDW forces with organic molecules due to similar molecular size, geometry, and component. Their large size and asymmetric geometry as well as the charge delocalization and charge transfer effects significantly weaken the electrostatic forces in ILs. As a result, the intermolecular force and vibrational force constant dominated by VDW interactions characterize the organic nature in ILs: the geometry near the minimum of the CEL (curvature and slope corresponding to force and force constant, respectively) are similar to polar organic solvents rather than inorganic molten salts. On the other hand, the cage energy, controlled by electrostatic interactions, characterizes the ionic nature in ILs: the depth of the CEL (cage energy) is apparently deeper than organic solvents. This cage mechanism, illustrated in Fig. 6, well explains the fact that though ILs show many similarities to organic solvents, such as melting temperature, solvation ability, and surface tension, they retain many distinctive properties, for example stability, non-volatility, and transport properties, apart them from organic solvents.

Generally, a larger cage energy (characterizing the liquid structure) corresponds to a higher activation energy (characterizing the liquid dynamics). In our cases, we have found that the cage energy has the right trend with the activation energy estimated from experimental diffusion data: [BMIM][PF_4_] > [BMIM][BF_4_] > [BMIM][NTf_2_] > DMSO > toluene (see the SI for the details). Note that [BMIM][BF_4_] has a smaller cage energy than other ILs due to the reduced ionic charges in its model. Molten NaCl has a lower cage energy than ILs but a comparable activation energy because of its high simulated temperature as well as its small and symmetric volume, which significantly smooths its CEL.

## Discussion

Liquid molecules spontaneously form cage structures with a certain local order (e.g. charge order in ILs and translational order in molecular liquids) extending to several coordination shells. The central molecule vibrates near the minimum of the CEL and frequently escapes the cage to diffuse. As shown in Fig. 6, the organic nature is reflected by the slope and curvature of the CEL, whereas the ionic nature is described by the depth of the CEL.

Previously, Kirchner *et al*.^22^ found that the VDW interaction plays an important role in determining the IL ion-pair energy landscape in the gas phase. Our work demonstrates that even in the liquid phase, the VDW interaction still plays an important role in determining the slope and curvature of the CEL of ILs, which explains the result reported by Balasubramanian and coworker^77^ that low-frequency vibrational modes in ILs are dominated by short-range interactions.

Our proposed microscopic mechanism provides a general way for understanding and predicting the unique properties of ILs. For example, the high viscosity and the non-volatility in ILs can be understood by their deep CEL, and the similarity between the CEL minima of ILs and organic solvents explains the fact that the IL surface tension is in the region for organic solvents rather than strong ionic fluids^48^. We can further predict more generally for any liquids that the properties relating to the CEL minimum will show more organic nature, whereas those corresponding to the CEL depth will display more ionic nature.

This work clarifies the blurry dual ionic and molecular nature of ILs and the corresponding microscopic mechanism provides a new insight into their unique properties. Our simulations pave the way for future experimental studies on complex interactions in liquids and our proposed CEL picture are hopeful to be verified by future experiments. The CEL concept proposed in the present paper is expected to advance our knowledge on complex liquids and to help better functionalization and industrial applications of various kinds of liquids.

## Methods

### Force field

The force field (FF) parameters for NaCl, DMSO, and toluene were all taken from the AMBER FF^78^, and the partial charges of DMSO and toluene were adopted from refs^50^,^51^, respectively. The parameters of IL [BMIM][BF_4_] were taken from the model developed by Wang *et al*.^79^ based on the AMBER force field and all the partial charges were scaled by a factor of 0.807, as suggested by Chaban *et al*.^49^, to incorporate the polarizability and charge transfer effects. For other ILs, the parametes were mainly taken from the AMBER FF (see ref. 20 for details). For molten NaCl, although the widely used Born-Mayer-Huggins potential with the Tosi-Fumi parameters^80^ may reproduce experimental results better than the AMBER FF, in order to make a direct comparison, in this work, we still use the AMBER FF to model molten NaCl and the quantitative differences caused by the AMBER FF will not change our qualitative conclusions.

Although more accurate FFs (e.g. Polarizable FF) and methods (such as ab initio MD and Car-Parrinello MD) may improve the simulation results quantitatively, they are still too expensive for simulating ILs, for which large system size and long simulation time are necessary. Since our simulation results have demonstrated that the empirical FFs can well reproduce the experimental results of ILs, we believe they are good enough to capture the qualitative features we are interested in.

### System setup

All the simulated systems contain 512 ion pairs or molecules in a cubic box with the periodic boundary condition applied to all three dimensions. A cutoff distance of 12 angstrom was applied to the VDW and the real part of the electrostatic interactions, and the particle-mesh Ewald method^81^ was employed to calculate the electrostatic interactions. In the equilibration procedures (simulated annealing and constant *NPT* simulations) the temperature and pressure were kept constant by using the Berendsen thermostat with a time constant of 0.1 ps and the Berendsen barostat with a time constant of 1 ps, respectively. In the production runs (constant *NVT* simulations) the temperature was kept constant by using the Nosé-Hoover thermostat with a time constant of 0.1 ps. All the simulations were performed by using the GROMACS software^82^ with a time step of 1 fs.

### Simulation details

The initial configuration of ILs were taken from our previous work^20^. For organic solvents, random configurations were initially equilibrated in a constant *NVT* ensemble by a simulated annealing procedure with five sequential steps: 200 ps at the temperature *T* = 2000 K, 300 ps at 1500 K, 500 ps at 1000 K, 1 ns at 700 K, and 1 ns at 500 K. For molten NaCl, a simulated annealing procedure was performed as: 200 ps at 2500 K, 300 ps at 2000 K, 500 ps at 1500 K, 1 ns at 1250 K, and 1 ns at 1150 K. A constant *NPT* simulation was then carried out at the pressure *P* = 1 atm for 3 ns for all the systems to determine the equilibrium simulation box size. With the determined box size, the constant *NVT* production runs were performed for 10 ns for ILs and 2 ns for other liquids. In these *NPT* and *NVT* runs, the temperature was kept constant at 1148.15 K for NaCl and 300 K for other systems. For all liquids, totally 2000 configurations were evenly sampled during the production runs.

### Quantum chemical calculation

The geometry optimizations were performed with the Gaussian 03 software^83^ at the HF/6-31G** level. The interionic interaction energy between an isolated gas-phase [BMIM][BF_4_] ion pair was calculated at the MP2/6-31G** level. The basis-set superposition error (BSSE) was corrected by using the counterpoise method.

### Calculation of intermolecular force

The force and its electrostatic and VDW parts experienced by each atom were sampled from MD simulations. The intermolecular forces were obtained by summing over all atomic forces experienced by each molecule.

### Calculation of intrinsic electric field

In a detailed simulation work^84^, the electric field experienced by a probe molecule was sampled to simulate the real vibrational Stark effect spectroscopy experiment^71^. In this work, in order to describe the intermolecular electrostatic interactions in liquids, no probe molecule was added and the average electric field experienced by each molecule was directly calculated by


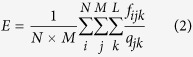


where *f* is the electrostatic force experienced by each atom, *q* is the atomic charge of each atom, *L* is the number of atoms in each molecule, *M* and *N* are the number of ions in the system and the number of sampled configurations, respectively.

### Calculation of cage energy

The cage energy, defined as the average potential energy between a molecular pair in the cage, was calculated by using the following equation,


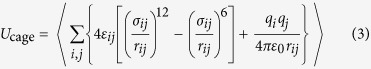


where 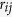 is the distance between atoms *i* and *j, q* is the atomic partial charge, 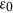 is the vacuum permittivity, and 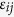 and 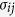 are the VDW parameters. *i, j* run over all atoms in a molecular pair, respectively, and 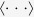 denotes the ensemble average.

## Additional Information

**How to cite this article**: Shi, R. and Wang, Y. Dual Ionic and Organic Nature of Ionic Liquids. *Sci. Rep.* **6**, 19644; doi: 10.1038/srep19644 (2016).

## Supplementary Material

Supplementary Information

## Figures and Tables

**Figure 1:**
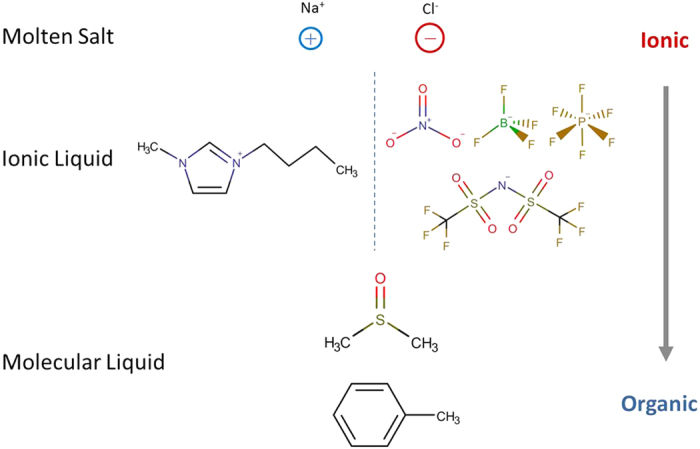
Molecular structures. From top to bottom: sodium chloride (NaCl), 1-butyl-3-methylimidazolium nitrate ([BMIM][NO_3_]), 1-butyl-3-methylimidazolium tetrafluoroborate ([BMIM][BF_4_]), 1-butyl-3-methylimidazolium hexafluorophosphate ([BMIM][PF_6_]), 1-butyl-3-methylimidazolium bis(trifluoromethanesulfonyl)imide ([BMIM][NTf_2_]), dimethyl sulfoxide (DMSO), and toluene, in such an order that the molecular feature changes from more ionic to more organic.

**Table 1:** Physicochemical properties.

	*T*_m_/K	*M*/g·mol^−1^	*ρ*/g·cm^−3^	*μ*/D	*γ*/mNm^−1^	*η*/mPas	*D*_cation_/10^−9^m^2^·s^−1^	*D*_anion_/10^−9^m^2^·s^−1^
NaCl	1073.15^33^	58.44	1.52^34^ (1.28)	—	108.6^34^	0.891^34^	10.7^35^ (7.3)	7.6^35^ (5.4)
[BMIM] [NO_3_]	309.2^36^	201.2	1.16^c^^37^ (1.17)	—	—	165.3^c^^37^	−(0.0009)	−(0.0004)
[BMIM] [BF_4_]	188.15^a^^38^	226.03	1.17^39^ (1.15)	—	40.4^40^	90.04^41^	0.016^41^ (0.016)	0.015^41^(0.010)
[BMIM] [PF_6_]	283.15^41^	284.2	1.37^41^(1.36)	—	45.7^40^	228.8^41^	0.008^41^ (0.0013)	0.006^41^ (0.0005)
[BMIM] [NTf_2_]	270.15^41^	419.4	1.44^41^ (1.51)	—	33.1^42^	45.7^41^	0.03^41^ (0.002)	0.024^41^ (0.002)
DMSO	291.65^43^	78.13	1.10^b^^44^ (1.11)	3.96^43^ (5.05)	43.0^b^^44^	1.97^b^^44^	0.7^c^^45^ (0.6)	
Toluene	178.15^43^	92.14	0.86^b^^44^ (0.83)	0.35^46^ (0.34)	28.5^b^^44^	0.56^b^^44^	2.4^47^ (2.4)	

Melting temperatures *T*_*m*_, molar masses *M*, densities *ρ*, dipole moments *μ*, surface tensions *γ*, viscosities *η*, and self-diffusion coefficients *D*. The values in parentheses represent our simulation results. Unless otherwise noted, all the values (except *T*_*m*_) for NaCl are obtained at *T* = 1148.15 K, and *T* = 300 K for the other three liquids.

^a^Glass transition temperature. No crystallization of [BMIM][BF_4_] has been observed. ^b^Data measured at 297 K. ^c^Data measured at 298.15 K.

**Table 2:** Force constants and intrinsic electric fields.

	〈ω〉/cm^−1^	*k*/10^26^ s^−2^	*E*/10^8^ V/m	Δ*E*/10^8^ V/m
NaCl	162 (−)	9.3	87.8	67.0 (−)
[BMIM][NO_3_]	86 (−)	2.6	22.1	1.3 (2.3^d^)
[BMIM] [BF_4_]	82 (85^a^)	2.4	20.8	0 (0^d^)
[BMIM][PF_6_]	76 (78)	2.0	18.0	−2.8 (−1.0^d^)
[BMIM][NTf_2_]	76 (77)	2.0	17.9	−2.9 (−2.5^d^)
DMSO	77 (82^b^)	2.1	23.9	3.1 (2.9^d^)
Toluene	69 (66^c^)	1.7	16.7	−4.1 (−4.6^d^)

Mean strengths of characteristic frequency (first moment) 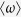, vibrational force constant *k*, intrinsic electric field *E*, and electric field difference Δ*E* with respect to [BMIM][BF_4_]. The values in parentheses are experimental ones.

^a^Calculated using data from ref. 68. ^b^Calculated using data from ref. 70. ^c^Calculated using data from ref. 69. ^d^Data from ref. 20,71.

**Table 3:** Cohesive energies.

	Δ_vap_*U*_m_	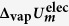	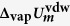	Δ_vap_*H*_m_
NaCl	233.9	248.8	−14.8	243.5 (219.2^a^)
[BMIM][NO_3_]	262.9	183.4	75.5	265.4 (−)
[BMIM][BF_4_]	127.2	72.5	61.3	129.7(128.2^b^)
[BMIM][PF_6_]	247.0	178.3	67.2	249.5 (−)
[BMIM][NTf_2_]	180.9	80.7	95.2	183.3 (155^73^)
DMSO	49.9	24.3	27.2	52.4 (52.9^c^)
Toluene	32.1	2.7	29.5	34.6 (38.0^d^)

Cohesive energy Δ_vap_*U*_m_, its electrostatic part 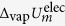
and VDW part 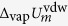, and heat of vaporization Δ_vap_*H*_m_. The experimental values are in the parentheses. The Unit is kJ/mol.

^a^Data at 1173.15 K from ref. 74.

^b^Data at 298.15K from ref. 75.

^c^Data at 300 K from ref. 50.

^d^Data at 298.15 K  K from ref. 76.

**Table 4:** Cage energies.

	*U*_cage_	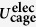	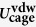
NaCl	−453.7	−464.5	10.8 (12.4, −1.6)
[BMIM][NO_3_]	−217.4	−213.9	−3.5 (7.9, −11.4)
[BMIM][BF_4_]	−162.8	−159.3	−3.5 (7.2, −10.7)
[BMIM][PF_6_]	−238.4	−233.8	−4.6 (8.9, −13.5)
[BMIM][NTf_2_]	−226.4	−215.6	−10.8 (13.0, −23.8)
DMSO	−7.2	−3.6	−3.6 (4.0, −7.6)
Toluene	−4.5	−0.4	−4.1 (3.1, −7.2)

Cage energy *U*_cage_, its electrostatic part 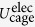 and VDW part 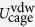. The VDW interaction was decomposed into the repulsive and attractive parts, respectively, as listed in the parentheses. The Unit is kJ/mol.
