# Supplementary material for: Dual Ionic and Organic Nature of Ionic Liquids
Source: Sci Rep. 2016 Jan 19;6:19644. doi: 10.1038/srep19644 (PMC4726069; doi:10.1038/srep19644)
Supplement: Supplementary Information [file srep19644-s1.pdf]

Supplementary Information

Dual Ionic and Organic Nature of Ionic Liquids

Rui Shi and Yanting Wang*

State Key Laboratory of Theoretical Physics, Institute of Theoretical Physics, Chinese Academy of Sciences, 55 East Zhongguancun Road, Beijing 100190 China

*Phone: +86 10-62648749. E-mail: wangyt@itp.ac.cn.

A. Vibrational density of state

The vibrational density of state (VDOS) can be calculated from the Fourier transform of velocity time autocorrelation by[1]

$$I(\omega) = \frac{1}{k_{\mathrm{B}}T} \sum_i m_i \frac{1}{2\pi} \int_{-\infty}^{\infty} \mathrm{d}t \exp(\text{-}i\omega t) \langle v_i(t) v_i(0) \rangle \tag{1}$$

where m is the mass, k_B is the Boltzmann constant, $v_i(t)$ is the velocity of atom type i at time t, and the angular brackets denote an average over time and all molecules (ions). All of the spectra are normalized by the area in the low-frequency region,

$$I_{\mathrm{norm}}(\omega) = I(\omega) \Big/ \int I(\omega) \mathrm{d}\omega \tag{2}$$

where $I(\omega)$ is the frequency-dependent intensity and $I_{\mathrm{norm}}(\omega)$ represents the area-normalized intensity.

The calculated VDOS was compared with optical heterodyne-detected Raman-induced Kerr effect spectroscopy (OHD-RIKES) at low frequencies, as shown in Figure S1. The OHD-RIKES of [BMIM][BF_4],[2] [BMIM][PF_6],[3] [BMIM][NTf_2],[3] DMSO,[4] and toluene[5] were obtained from experiments. The OHD-RIKES spectra of molten NaCl and [BMIM][NO_3] are not available in the literature. It can be seen that the calculated VDOSs are able to capture the main features of the experimental OHD-RIKES. Since the VDOS is weighted by mass, whereas the OHD-RIKES signal is weighted by atom polarization,[6] the calculated peak heights differ from the experimental spectra.

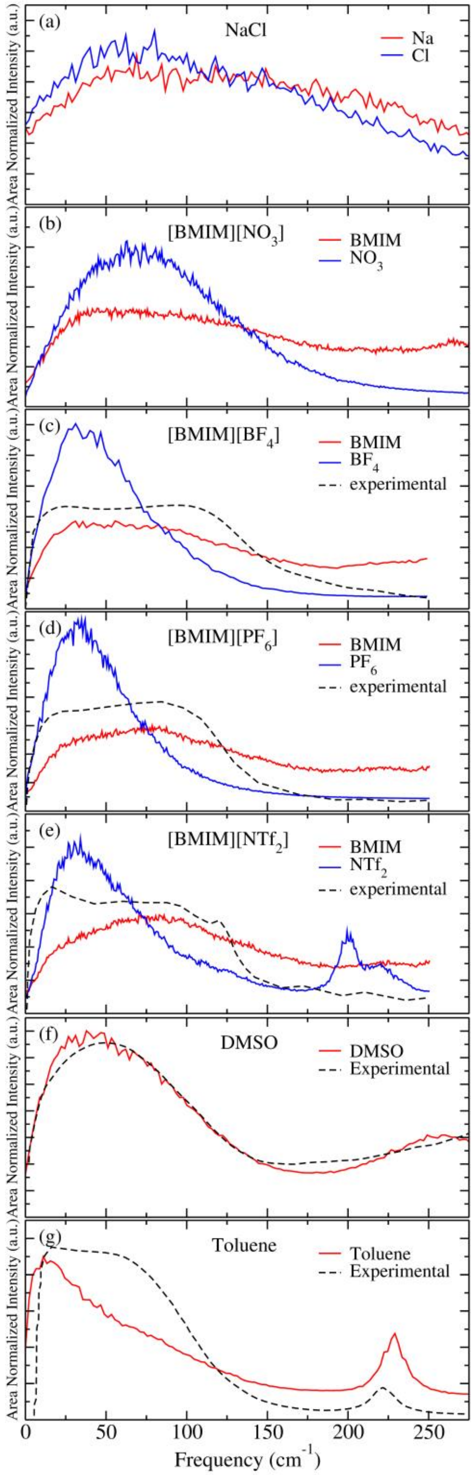

Figure S1. Vibrational density of states and optical heterodyne-detected Raman-induced Kerr effect spectroscopy at low frequencies for molten (a) NaCl, (b) [BMIM][NO_3], (c) [BMIM][BF_4], (d) [BMIM][PF_6], (e) [BMIM][NTf_2], (f) DMSO, and (g) toluene.

B. Ion pair and ion cage

In Figure S2, we plot the distributions of the distance between cation and anion in an ion pair in the gas phase, in an ion pair in the liquid state, and in an ion cage in IL [BMIM][BF_4], respectively. The ion-pair distance in the gas phase was determined by the quantum chemical calculation, and the ion-pair distance in the liquid phase and ion cage were calculated from the molecular dynamics simulations. It can be seen that the most-probable distance between cation and anion is about 3.5 Å for a gas-phase ion pair, 4.3 Å for a liquid-state ion pair, and 4.8 Å for the ion cage.

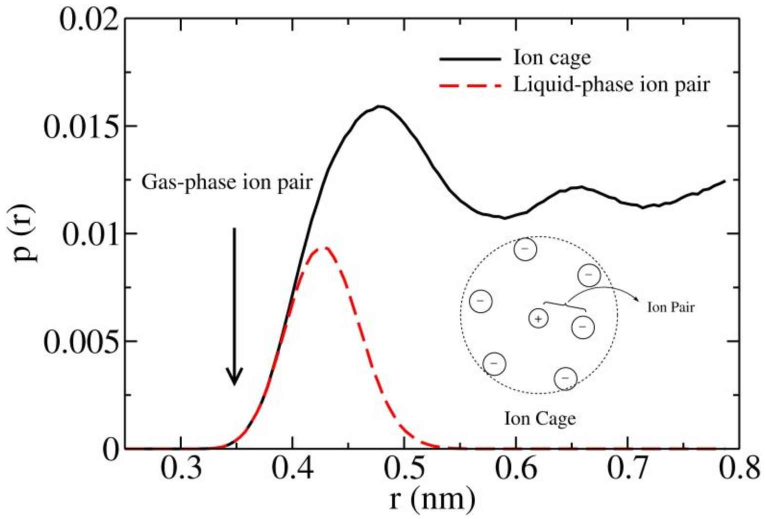

Figure S2. Distributions of the distance between cation and anion in IL [BMIM][BF_4]. Arrow: gas-phase ion pair; black solid line: liquid-phase ion cage; red dash line: liquid-phase ion pair. Inset: schematic illustration of the liquid-phase ion pair and ion cage in ILs.

C. Binding energy

The binding energy of an ion pair in the gas phase has been widely studied by *ab initio* calculations for different ILs.[7, 8, 9, 10, 11, 12, 13, 14] Due to the limitation of computer power, only an isolated ion pair or small ion clusters in the gas phase were investigated in those calculations. In

the present work, following the methodology of Tsuzuki,[15] we calculated the binding energy of a [BMIM][BF_4] ion pair at the MP2/6-31G** level with the BSSE correction. A very high interaction energy of 353.07 kJ/mol was obtained, close to the DFT results of 341.14 kJ/mol reported by Fumino[16] and 344.96 kJ/mol by Hunt.[17] We also calculated the interaction energy using the same optimized geometry by the AMBER force field. The interaction energy with the reduced-charge model (0.807e) was 219.75 kJ/mol, much smaller than 340.85 kJ/mol with the original model (1.0e) developed by Wang et al..[18] Even though Wang's model reproduces the gas-phase binding energy well, it apparently underestimates the system dynamics in the liquid phase. In contrast, by considering the polarizability and charge transfer effects in the liquid phase, the reduced-charge model reproduces both structural and dynamical properties of ILs very well.[19] By using the reduced-charge model of [BMIM][BF_4], the calculated cage energy of 162.8 kJ/mol is 26% smaller than the gas-phase binding energy of 219.75 kJ/mol, which can be explained by the fact that ILs have a local structure in the liquid phase different from in the gas phase, as shown in Fig. S2.

D. Relation between cage energy and activation energy

Cage energy, defined as the average potential energy between an ion and a counterion within its ion cage, describes the stability of a cage structure. It is related to the microscopic dynamics of the system: a lower cage energy makes the ions more difficult to escape from the cage and thus the dynamics becomes slower. From this point of view, the structure-based cage energy should somehow relate to the dynamics-based activation energy. According to the Arrhenius law, particles have to climb over an energy barrier to diffuse from one local minimum to another, and

the activation energy is the average energy that a particle needs to climb over the energy barrier. This activation process is described by the Arrhenius equation, $X = X_0 \exp\left(-E_a / k_B T\right)$, where X is a dynamic property, such as the diffusion coefficient, conductivity, or inverse of viscosity, X_0 is a constant, E_a is the activation energy, and k_B is the Boltzmann constant. By applying the Arrhenius law to the experimental diffusion data, we have estimated the activation energies for the four liquids and list them in Table S1. Since ILs usually disobey the Arrhenius law at low temperatures, we estimated the activation energies of ILs within a narrow room temperature range. (Data for [BMIM][NO_3] was not found.) It can be seen that, except molten NaCl, the liquid with a lower cage energy has a higher activation energy, because molecules have to overcome the cohesive cage energy to climb over the energy barrier. Moreover, dynamics in liquids is usually quite collective: proper rearrangement of nearby ions is required to allow a single diffusive motion. Besides the cage energy, ions in ILs have to diffuse by overcoming the strong steric hindrance induced by the large volume and asymmetric geometry of surrounding organic ions, which significantly increases the activation energy of ILs. Therefore, [BMIM][BF_4] has an activation energy as high as molten NaCl, even though its cage energy is much lower than molten NaCl.

Table S1 | Cage energy and activation energy. The activation energy for self-diffusion $E_a^{\pm}$ was obtained by fitting the experimental data with the Arrhenius law. The unit is kJ/mol.

	U_{cage}	E_a^+	E_a^-
NaCl	-453.7	32.9[a]	35.1[a]
[BMIM][BF_4]	-162.8	32.5[b]	34.4[b]
[BMIM][PF_6]	-238.4	37.6[b]	39.1[b]
[BMIM][NTf_2]	-226.4	27.6[b]	28.7[b]
DMSO	-7.2	19.0[c]	
Toluene	-4.5	10.6[d]	

[a]Fitting the diffusion coefficient data in the temperature range of 1103.15 to 1263.15 K from Ref. [20].

[b]Fitting the diffusion coefficient data in the temperature range of 303 to 328 K from Ref. [21].
[c]Fitting the diffusion coefficient data in the temperature range of 273.0 to 320.0 K from Ref. [22].
[d]Fitting the diffusion coefficient data in the temperature range of 273.5 to 325.65 K from Ref. [23].

REFERENCES

1. Berne BJ, Pecora R. *Dynamic Light Scattering*. John Wiley (1976).
2. Lu R, Wang W, Yu A. Cation and anion substitution effects on the ultrafast dynamics of interionic interaction in imidazolium based ionic liquids. *Sci China-Chem* **54**, 1491-1497 (2011).
3. Giraud G, Gordon CM, Dunkin IR, Wynne K. The effects of anion and cation substitution on the ultrafast solvent dynamics of ionic liquids: A time-resolved optical Kerr-effect spectroscopic study. *J Chem Phys* **119**, 464-477 (2003).
4. Wiewiór PP, Shirota H, Castner EW. Aqueous dimethyl sulfoxide solutions: Inter- and intra-molecular dynamics. *J Chem Phys* **116**, 4643-4654 (2002).
5. Shirota H. Ultrafast molecular dynamics of liquid aromatic molecules and the mixtures with CCl_4. *J Chem Phys* **122**, 044514 (2005).
6. Hu Z, Huang X, Annapureddy HVR, Margulis CJ. Molecular Dynamics Study of the Temperature-Dependent Optical Kerr Effect Spectra and Intermolecular Dynamics of Room Temperature Ionic Liquid 1-Methoxyethylpyridinium Dicyanoamide. *J Phys Chem B* **112**, 7837-7849 (2008).
7. Bernard UL, Izgorodina EI, MacFarlane DR. New Insights into the Relationship between Ion-Pair Binding Energy and Thermodynamic and Transport Properties of Ionic Liquids. *J Phys Chem C* **114**, 20472-20478 (2010).
8. Dong K, Song Y, Liu X, Cheng W, Yao X, Zhang S. Understanding Structures and Hydrogen Bonds of Ionic Liquids at the Electronic Level. *J Phys Chem B* **116**, 1007-1017 (2011).
9. Fernandes AM, Rocha MAA, Freire MG, Marrucho IM, Coutinho JAP, Santos LMNBF. Evaluation of Cation−Anion Interaction Strength in Ionic Liquids. *J Phys Chem B* **115**, 4033-4041 (2011).
10. Izgorodina EI, Bernard UL, MacFarlane DR. Ion-Pair Binding Energies of Ionic Liquids: Can DFT Compete with Ab Initio-Based Methods? *J Phys Chem A* **113**, 7064-7072 (2009).
11. Köddermann T, Wertz C, Heintz A, Ludwig R. Ion-Pair Formation in the Ionic Liquid 1-Ethyl-3-methylimidazolium Bis(triflyl)imide as a Function of Temperature and Concentration. *ChemPhysChem* **7**, 1944-1949 (2006).
12. Ludwig R, Paschek D. Applying the Inductive Effect for Synthesizing Low-Melting and Low-Viscosity Imidazolium-Based Ionic Liquids. *ChemPhysChem* **10**, 516-519 (2009).
13. Turner EA, Pye CC, Singer RD. Use of ab Initio Calculations toward the Rational Design of Room Temperature Ionic Liquids. *J Phys Chem A* **107**, 2277-2288 (2003).
14. Zhang S, Qi X, Ma X, Lu L, Zhang Q, Deng Y. Investigation of cation–anion interaction in 1-(2-hydroxyethyl)-3-methylimidazolium-based ion pairs by density functional theory calculations and experiments. *J Phys Org Chem* **25**, 248-257 (2012).

15. Tsuzuki S, Tokuda H, Hayamizu K, Watanabe M. Magnitude and Directionality of Interaction in Ion Pairs of Ionic Liquids: Relationship with Ionic Conductivity. *J Phys Chem B* **109**, 16474-16481 (2005).
16. Fumino K, Wulf A, Ludwig R. The potential role of hydrogen bonding in aprotic and protic ionic liquids. *Phys Chem Chem Phys* **11**, 8790-8794 (2009).
17. Hunt PA, Gould IR, Kirchner B. The Structure of Imidazolium-Based Ionic Liquids: Insights From Ion-Pair Interactions. *Aust J Chem* **60**, 9-14 (2007).
18. Liu Z, Huang S, Wang W. A Refined Force Field for Molecular Simulation of Imidazolium-Based Ionic Liquids. *J Phys Chem B* **108**, 12978-12989 (2004).
19. Chaban VV, Voroshylova IV, Kalugin ON. A new force field model for the simulation of transport properties of imidazolium-based ionic liquids. *Phys Chem Chem Phys* **13**, 7910-7920 (2011).
20. Bockris JOM, Hooper GW. Self-diffusion in molten alkali halides. *Discuss Faraday Soc* **32**, 218-236 (1961).
21. Tokuda H, Hayamizu K, Ishii K, Susan MABH, Watanabe M. Physicochemical Properties and Structures of Room Temperature Ionic Liquids. 1. Variation of Anionic Species. *J Phys Chem B* **108**, 16593-16600 (2004).
22. Packer KJ, Tomlinson DJ. Nuclear spin relaxation and self-diffusion in the binary system, dimethylsulphoxide (DMSO)+ water. *Trans Faraday Soc* **67**, 1302-1314 (1971).
23. Harris KR, Alexander JJ, Goscinska T, Malhotra R, Woolf LA, Dymond JH. Temperature and density dependence of the selfdiffusion coefficients of liquid n-octane and toluene. *Mol Phys* **78**, 235-248 (1993).
